# Vibronic Dynamics of Photodissociating ICN from Simulations of Ultrafast X‐Ray Absorption Spectroscopy

**DOI:** 10.1002/anie.202007192

**Published:** 2020-08-31

**Authors:** Uriel N. Morzan, Pablo E. Videla, Micheline B. Soley, Erik T. J. Nibbering, Victor S. Batista

**Affiliations:** ^1^ Condensed Matter Section The Abdus Salam International Center for Theoretical Physics Strada Costiera 11 34151 Trieste Italy; ^2^ Department of Chemistry Yale University P.O. Box 208107 New Haven CT 06520-8107 USA; ^3^ Energy Sciences Institute Yale University P.O. Box 27394 West Haven CT 06516-7394 USA; ^4^ Max Born Institute for Nonlinear Optics and Short Pulse Spectroscopy Max Born Strasse 2A 12489 Berlin Germany; ^5^ Department of Chemistry and Chemical Biology Harvard University 12 Oxford Street Cambridge MA 02138 USA; ^6^ Yale Quantum Institute Yale University P.O. Box 208334 New Haven CT 06520-8263 USA

**Keywords:** ICN, pump–probe spectroscopy, quantum dynamics, time-resolved X-ray spectroscopy, ultrafast photochemistry

## Abstract

Ultrafast UV‐pump/soft‐X‐ray‐probe spectroscopy is a subject of great interest since it can provide detailed information about dynamical photochemical processes with ultrafast resolution and atomic specificity. Here, we focus on the photodissociation of ICN in the ^1^Π_1_ excited state, with emphasis on the transient response in the soft‐X‐ray spectral region as described by the ab initio spectral lineshape averaged over the nuclear wavepacket probability density. We find that the carbon K‐edge spectral region reveals a rich transient response that provides direct insights into the dynamics of frontier orbitals during the I−CN bond cleavage process. The simulated UV‐pump/soft‐X‐ray‐probe spectra exhibit detailed dynamical information, including a time‐domain signature for coherent vibration associated with the photogenerated CN fragment.

## Graphical abstract

Ultrafast UV‐pump/soft‐X‐ray‐probe spectroscopy provides detailed information about dynamical photochemical processes with ultrafast resolution and atomic specificity. Here, the photodissociation of ICN in the ^1^Π_1_ excited state is studied. The carbon K‐edge spectral region reveals a rich transient response that provides direct insights into the dynamics of frontier orbitals during the I−CN bond cleavage process.

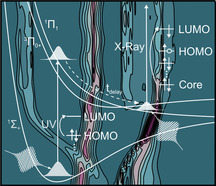

## Introduction

In this study, we explore the potential of ultrafast UV‐pump/soft‐X‐ray‐probe spectroscopy to gain fundamental understanding of dynamical photochemical processes with atomic specificity and ultrafast resolution. Ultrafast laser technology has revolutionized the field of chemistry by enabling powerful techniques to monitor elementary steps of chemical reactions.^[1]^ Pioneering UV/Vis electronic pump–probe studies of small molecules in the gas‐phase, including the influential work of Zewail and co‐workers,^[1c]^ have provided valuable insights into the interplay between vibrational modes and chemical bond rearrangements. Photoinduced chemical reactions in solution have also been studied extensively,^[2]^ where solute‐solvent couplings and spectral broadening typically limited the amount of structural information that could be obtained from a cursory examination of the spectroscopic signals. Ultrafast vibrational spectroscopy allows probing specific vibrational modes of chemical bonds in transient species, which provides dynamical information about structural rearrangements.[^2a^, ^2d^] For delocalized vibrational mode patterns, transient changes in the electronic structure can only be obtained in terms of quantum chemical calculations of the corresponding transient electronic states.^[3]^

Recent developments in ultrafast X‐ray spectroscopy open new opportunities to probe changes in transient electronic structure during the course of a chemical reaction. Ultrafast X‐ray spectroscopies,^[4]^ including X‐ray absorption spectroscopy (XAS),[^3a^, ^5^] X‐ray emission spectroscopy (XES), and resonant inelastic X‐ray scattering (RIXS) can enable a direct characterization of the valence electronic structure dynamics.[^6^, ^7^]

Recent results probing occupied (and unoccupied) orbitals of organic and organometallic systems have provided key insights into the electronic structural rearrangements of a variety of molecular systems undergoing photoinduced bond‐breaking reactions.[^2d^, ^2e^, ^8^] In this study, we explore the capabilities of ultrafast UV‐pump/soft‐X‐ray‐probe spectroscopy to monitor the underlying dynamics of frontier molecular orbitals during the photolysis reaction of ICN.^[9]^

**Figure 1:**
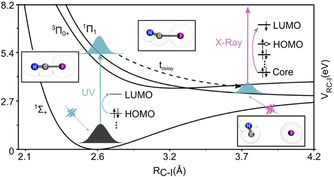
The pump–probe UV/X‐ray spectroscopy employed in this study as applied to the photodissociation dynamics of the ICN molecule. A UV pulse photoexcites the initial wavepacket according to the ^1^Π_1_←^1^Σ^+^ transition, while a delayed carbon K‐edge X‐ray pulse probes the transient vibronic dynamics. The figure illustrates the potential energy surface along the C−I bond, where the photodissociation dynamics involves the breaking of the C−I bond coupled to the rotational motion of the photogenerated CN fragment.

Photoexcitation of the ICN continuum band with an ultraviolet pump pulse induces ultrafast dissociation of ICN into I and CN fragments (Figure 1). Previous studies have determined that mainly two electronic states are involved in the dissociation paths:^[10]^ the ^3^Π_0+_ state that forms the I*(^2^ *P*_1/2_) fragment, and the ^1^Π_1_ state that correlates with the high energy range of the band and dissociates into the I(^2^ *P*_3/2_) fragment.[^9a^, ^9e^] The two photofragmentation pathways branch out at a conical intersection with branching ratios that change as a function of the photoexcitation wavelength, as in many other photochemical reactions.^[11]^

In this study we focus on photoexcitation from the ground state to the ^1^Π_1_ state with negligible population transfer to the ^3^Π_1_ state. We simulate the photoinduced transient dynamics in the excited state to investigate the description that could be obtained from UV‐pump/soft‐X‐ray‐probe spectroscopy using wavepacket propagation on accurate potential energy surfaces[^9a^, ^9e^] and an electronic response evaluated with a simple approach based on a combination of the maximum overlap method (MOM)^[12]^ and configuration interaction singles (CIS)^[13]^ (see the Supporting Information). While our approach could also be implemented with more accurate electronic structure schemes,[^3h^, ^3i^] we show the method provides the fundamental aspects of ICN photodissociation dynamics that could be probed by UV‐pump/soft‐X‐ray‐probe spectroscopy.

Our findings indicate that the femtosecond time resolution and atomic specificity of soft‐X‐ray spectroscopy[^5^, ^21^] enable a detailed molecular movie of the ICN photofragmentation dynamics to be captured, including the production of vibrationally hot CN fragments along the I−C dissociation path during the ultrafast relaxation dynamics on the ^1^Π_1_ excited electronic state. Furthermore, we show the spectral signature can provide an unequivocal interpretation of the changes in bond‐order parameters induced by transient electronic structure rearrangements responsible for the photodissociation.

## Results and Discussion

**Figure 2:**
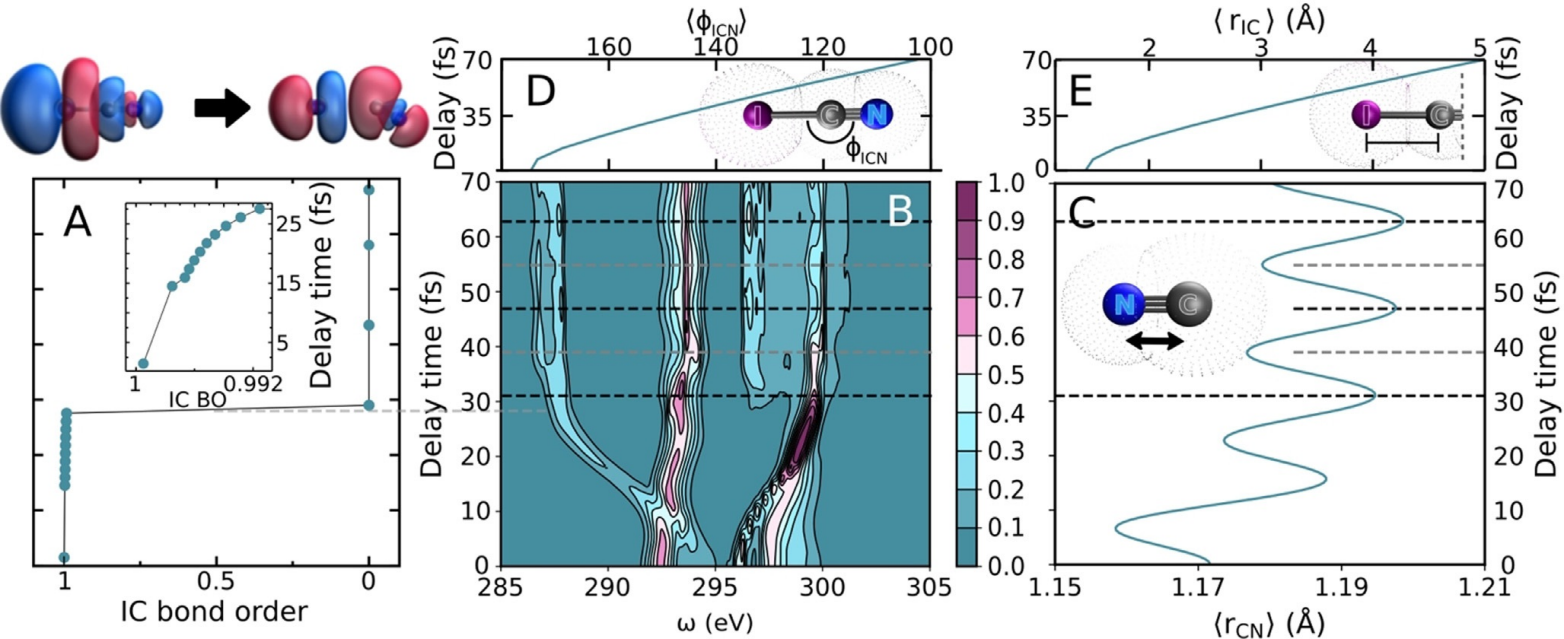
A) Time‐dependent bond order of the dissociating I−C bond, computed as a function of time during the photodissociaton dynamics in the ^1^Π_1_ excited state probed by simulated UV/X‐ray spectroscopy. B) Carbon K‐edge transient X‐ray spectrum of ICN following ^1^Π_1_←^1^Σ^+^ photoexcitation by the UV pulse. C) Time‐dependent C−N bond length during the ICN photodissociation. Dashed lines indicate the time of amplitude maxima (gray) and minima (black). D),E) Time‐dependent ICN angle and I−C bond length during the ICN photodissociation dynamics.

Figure 2 shows the predicted capabilities of UV‐pump/soft‐X‐ray‐probe spectroscopy for characterization of time‐dependent changes in the electronic structure of ICN triggered by photoexcitation to the ^1^Π_1_ state. The transient X‐ray absorption spectrum (TRXAS) in the C K‐edge region was obtained by averaging the spectral lineshape over the nuclear wavepacket probability density (see the Supporting Information).[^3e^, ^14^]

The computed transient spectrum (Figure 2 B) provides clear fingerprints of the nuclear and electronic dynamics in the ^1^Π_1_ excited state. The lower energy region of the spectrum is characterized at early times by two broad peaks that branch out into three bands within about 30 fs. Vibronic oscillations are observed after about 20 fs in the central and higher frequency bands. The oscillations correspond to the C−N vibration, as shown by the agreement between the time‐dependent modulation of intensities in the TRXAS and the time‐dependent expectation value of the C‐N distance ⟨*r*_CN_⟩ (Figure 2 C). The oscillation period is about 16 fs (ca. 2085 cm^−1^), in close agreement with the vibrational frequency of the CN radical.^[15]^ These coherences will be revealed in the experimental TRXAS spectrum so long as the durations of the pump and probe pulses do not exceed the CN stretching period.

Of note, the CN vibrations are also exhibited in the N K‐edge TRXAS (Supporting Information, Figure S1), although just weakly observed in the I K‐edge, which reflects atomic specificity as an important advantage offered by ultrafast UV‐pump/soft‐X‐ray‐probe spectroscopy. Given the non‐bonded nature of the 1s core electrons, the K‐edge absorption bands are typically separated by tens or hundreds of eV for different atoms. The energy separation enables one to probe specific atoms, with pulses tuned at their corresponding frequencies, to monitor the local dynamics of individual fragments in the system. In this context, we find that UV/X‐ray pump‐probe spectroscopy can provide unique insights into the nuclear motion of specific vibrations, as demonstrated here for the ultrafast relaxation dynamics of ICN on the femtosecond time scale.

The I−C bond dissociation is clearly observed in the TRXAS (Figure 2 B). During the first 30 fs after UV photoexcitation the three bands in the spectrum display a significant frequency shift. In particular, the lower energy signal branches into two bands, one of which shows a very pronounced red‐shift of about 9 eV. These shifts are originated in the rotation of the CN fragment (Figure 2 D) and elongation of the I−C bond (Figure 2 E). Moreover, right before 30 fs, when the X‐ray absorption bands complete their branching process and reach their asymptotic values, there is a significant drop in the I−C bond order, consistent with the breaking of the I−C bond (Figure 2 A). This demonstrates that the TRXAS provides direct information about the nature of chemical bonding and the vibronic behavior of molecular fragments photogenerated in the sub‐100 femtosecond time scale.

It is worth mentioning that the energy resolution of the TRXAS, shown in Figure 2, is only limited by the intrinsic core–hole lifetime, whereas in an experiment the signal would have to be convoluted with the instrumental resolution. To estimate the minimum spectral resolution needed for experimental observation of such features, we convoluted the original spectrum with a Gaussian pulse of varying full width at half maximum (FWHM) (see the Supporting Information). We observe that unequivocal signs of both the I−C dissociation dynamics and the CN vibrational motions are retained by the broadened TRXAS with pulses of up to a FWHM of about 3 eV (Supporting Information, Figure S2). Spectral broadening with FWHM >4.5 V masks the dynamical information of the photodissociation process by merging all peaks into a broad band centered at 295 eV. Since a spectral resolution of ≤1.2 eV at the carbon K‐edge has already been reported for current laser‐based table‐top UV/X‐ray pump–probe setups (utilizing extreme high‐order harmonic radiation and with clear options for further improvements)^[16]^ and resolution at large scale facilities is about an order of magnitude better,[^2b^, ^17^] measuring the predicted oscillatory features should be clearly within experimental reach.

The near‐K‐edge condition enables interpretation of the TRXAS signals in terms of the ensuing dynamical evolution of frontier orbitals induced by UV photoexcitation. The natural transition orbitals (NTOs) of the carbon near‐K‐edge excitations of ICN in the ^1^Π_1_ excited state are the predominant components of the state occupied by the 1s core excitation. Hence, the NTOs provide a valuable description of the frontier orbitals occupied upon X‐ray absorption.

**Figure 3:**
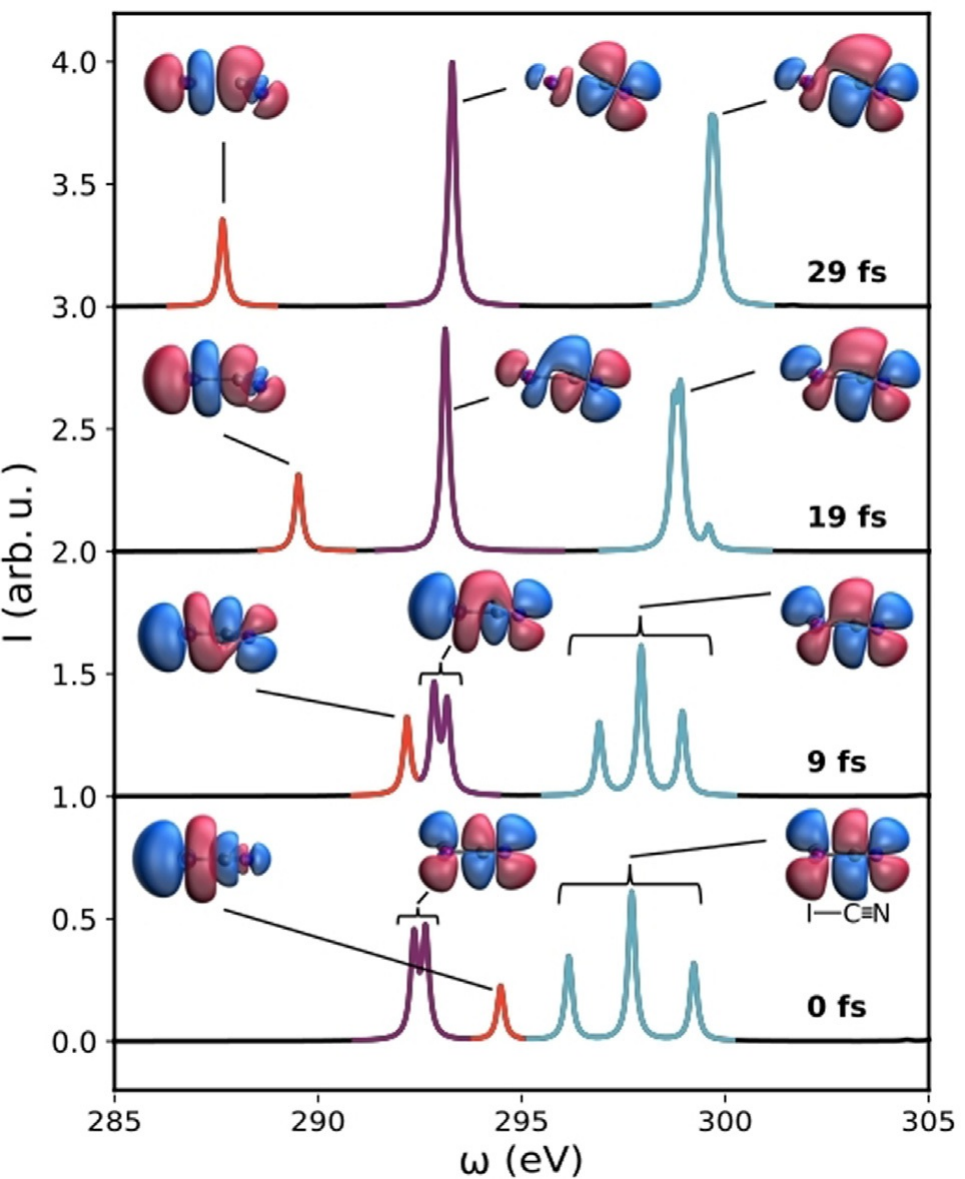
Carbon K‐edge X‐ray absorption spectra and natural transition orbitals (NTOs) associated with each group of transitions of ICN from the ^1^Π_1_ excited state at representative configurations during the early‐time photodissociation dynamics. Spectra at different times are shifted vertically for clarity.

To clearly visualize the rich information on the electronic structure dynamics that is encoded in the TRXAS, Figure 3 shows a simplified view of the spectral evolution, taking four representative configurations of ICN along the photodissociation pathway (which correspond to the expectation values of the nuclear coordinates obtained from the ICN time‐dependent wavepacket). Each peak is depicted with its corresponding NTO to establish a connection between the electronic rearrangements and the spectral changes.

The initial ICN configuration, corresponding to the ground state equilibrium geometry, or a pump‐probe delay time of 0 fs, reveals a XAS spectrum composed of π* antibonding frontier orbitals with X‐ray absorption energies in the 295–300 eV range and a σ* antibonding orbital at 298 eV (Figure 3, bottom). All of these orbitals are significantly affected by the photodissociation process, as evidenced by their energy shifts towards the asymptotic values at *t*>30 fs. The carbon 1s→σ* transition is distinct from the other peaks in the TRXAS since the σ* orbital has the symmetry of the dissociating I−C bond, which explains its higher sensibility to the dissociation process. The red‐shift associated with this transition is a consequence of the anti‐bonding I−C character. In contrast, during the photodissociation process, the CN rotation breaks the π* character of the carbon 1s→π* transitions, which evolve to be mainly localized on the CN photofragment. As a consequence, the carbon 1s→π* transitions experience a small blue shift. These results show that the XAS spectral shifts can be employed as a direct observable of the symmetry of frontier orbitals.

The vibronic oscillation associated with the CN photofragment is much more evident in the carbon 1s→π* transitions. The vibrating CN triple bond has both the π symmetry of the C1s→π* bands and the σ symmetry of the C1s→σ* transition and therefore exhibits significant oscillations that correlate with the CN vibration. As discussed above, the NTOs associated with the C1s→π* peaks are mainly localized on the vibrating CN fragment, with a very small delocalization on I, while for the C1s→σ* transitions the orbitals are spread over the entire ICN molecule. This is reflected in the higher sensitivity to the CN vibrations displayed by the C1s→π* bands in comparison with the C1s→σ* bands. Therefore, we find that the TRXAS provides valuable information on both the vibronic dynamics and the localization of the orbitals involved in the corresponding X‐ray absorption transitions.

## Conclusion

We have shown the capabilities of UV‐pump/soft‐X‐ray‐probe spectroscopy to monitor ultrafast nuclear dynamics and transient electronic structure rearrangements during the photodissociation of ICN in the ^1^Π_1_ excited state. Our findings suggest that currently available instrumental resolution with pulses with FWHM ≤1.2 eV of laser‐based table‐top UV/X‐ray pump‐probe setups should be sufficient to resolve the predicted oscillatory features and capture the molecular movie of the ultrafast photodissociation process, since the excited state dynamics can be clearly identified for a spectral broadening below about 3 eV. The present work reveals the potential of UV/soft‐X‐Ray pump‐probe spectroscopy to resolve the dynamics of the frontier orbitals responsible for chemical bonding and vibrational motions with atomic specificity. These unique capabilities should benefit the exploration of a plethora of chemical transformations, including conformational changes associated with isomerization reactions, bond dissociation, recombination and charge transfer processes.

Future work will be focused on the extension of the present computational scheme to analyze ultrafast dynamics in more complex systems. Although the use of exact wave‐packet propagation is unfeasible for condensed phases, despite recent numerical advances,^[18]^ the development of alternative dynamical representations, including semiclassical approximations^[19]^ and/or hybrid QM/MM Born‐Oppenheimer schemes,^[20]^ will enable exploration of the use of UV‐pump/soft‐X‐ray‐probe spectroscopy to elucidate chemical dynamics in condensed phases, such as biological systems or processes in solution. In particular, the development of proper modeling of rotational and vibrational cooling, geminate recombination, isomerization to iodoisocyanide (INC), hydrogen abstraction by the cyano radical fragment, and coherent control of ICN in solution[^9f^, ^9g^, ^21^] will be rich topics for further exploration.

## Conflict of interest

The authors declare no conflict of interest.

## Supporting information

As a service to our authors and readers, this journal provides supporting information supplied by the authors. Such materials are peer reviewed and may be re‐organized for online delivery, but are not copy‐edited or typeset. Technical support issues arising from supporting information (other than missing files) should be addressed to the authors.

Supplementary
