# Supplementary material for: Vibronic Dynamics of Photodissociating ICN from Simulations of Ultrafast X‐Ray Absorption Spectroscopy
Source: Angew Chem Int Ed Engl. 2020 Aug 31;59(45):20044–8. doi: 10.1002/anie.202007192 (PMC7693200; doi:10.1002/anie.202007192)
Supplement: Supplementary file 1 — Supplementary [file ANIE-59-20044-s001.pdf]

Angewandte Chemie
Eine Zeitschrift der Gesellschaft Deutscher Chemiker

Supporting Information

Vibronic Dynamics of Photodissociating ICN from Simulations of Ultrafast X-Ray Absorption Spectroscopy

Uriel N. Morzan[+], Pablo E. Videla[+],* Micheline B. Soley[+], Erik T. J. Nibbering, and Victor S. Batista**

anie_202007192_sm_miscellaneous_information.pdf

Supplementary Material - Vibronic Dynamics of Photodissociating ICN from Simulations of Ultrafast X-Ray Absorption Spectroscopy

Uriel N. Morzan, [a,b],†,* Pablo E. Videla,[b,c] ,†,* Micheline B. Soley, [b,e,f], † Erik T. J. Nibbering, [d] and Victor S. Batista [b,c], *

[a] Condensed Matter Section.
The Abdus Salam International Center for Theoretical Physics.
Strada Costiera 11, 34151. Trieste, Italy

[b] Department of Chemistry,
Yale University.
P.O. Box 208107, New Haven CT, 06520-8107, USA.

[c] Energy Sciences Institute,
Yale University.
P.O. Box 27394, West Haven, CT, 06516-7394, USA.

[d] Max Born Institute for Nonlinear Optics and Short Pulse Spectroscopy.
Max Born Strasse 2A, 12489 Berlin, Germany.

[e] Department of Chemistry and Chemical Biology,
Harvard University,
12 Oxford Street, Cambridge, MA, 02138, USA

[f] Yale Quantum Institute,
Yale University.
P. O. Box 208334, New Haven, CT 06520-8263, USA.

† Equally contributing authors.

* To whom correspondence should be addressed: umorzan@ictp.it, pablo.videla@yale.edu, victor.batista@yale.edu

Wavepacket propagation

The dissociation dynamics of ICN is modeled in terms of wavepacket propagation in the diabatic representation using Jacobi coordinates $\boldsymbol{R}$, $\boldsymbol{\theta}$ and $\boldsymbol{r}$, where $\boldsymbol{R}$ represents the distance between the I atom and the CN center of mass, $\boldsymbol{r}$ is the CN distance and $\boldsymbol{\theta}$ is the angle between the $\boldsymbol{R}$ and $\boldsymbol{r}$ vectors, as reported in earlier work.[9c] The Hamiltonian of the system restricted to move with total angular momentum J = 0 is

$$\widehat{\boldsymbol{H}}\,(\boldsymbol{R},\boldsymbol{r},\boldsymbol{\theta}) = \widehat{\boldsymbol{T}}(\boldsymbol{R},\boldsymbol{r},\boldsymbol{\theta}) + \widehat{\boldsymbol{V}}\,(\boldsymbol{R},\boldsymbol{r},\boldsymbol{\theta}) \quad (1)$$

where $\widehat{T}(R,r,\theta)$ is the kinetic energy approximated assuming a 2D rotor as follows:

$$\widehat{\boldsymbol{T}}(\boldsymbol{R},\boldsymbol{r},\boldsymbol{\theta}) \approx -\frac{1}{2\mu_{I,CN}}\frac{\partial^2}{\partial R^2} - \frac{1}{\mu_{CN}}\frac{\partial^2}{\partial r^2} - \frac{1}{2}\left[\frac{1}{\mu_{I,CN}R^2} + \frac{1}{\mu_{CN}r^2}\right]\frac{\partial^2}{\partial\theta^2} \quad (2)$$

with $\mu_{I,CN}$ and μ_{CN} representing the reduced masses of the I-CN and CN fragments, respectively. The potential $\widehat{V}\,(R,r,\theta)$ was modeled using the *ab initio* potential energy surfaces developed by Morokuma *et al.* [9a] and included the diabatic terms that correlate to the $^1\Pi_1$ (A') and $^3\Pi_{0+}$ (A') excited states (terms V_1 and V_3, respectively, in Ref. [9a]) and their interstate coupling (V_{13}). Note that the A'' component of the $^1\Pi_1$ does not mix with A' components and was therefore excluded from the dynamics. [9a] The $^3\Pi_1$ (A',A'') states were omitted since they do not couple significantly to the other states and are negligibly populated when the high energy range of the $\tilde{A}$ band is photo-excited (the ratio of oscillator strength transitions from the ground state to $^3\Pi_{0+}$, $^1\Pi_1$, and $^3\Pi_1$ is 0.66:0.28:0.06[9a]). Note that since the potentials are defined in terms of a different set of coordinates $\tilde{R}$ and $\tilde{\theta}$ (where $\tilde{R}$ is the distance between the I and C atoms, $\tilde{\theta}$ is the angle between the $\tilde{R}$ and r vectors), a coordinate transformation based on the law of cosines was applied to compute the potential in terms of Jacobi coordinates.

The initial wavefunction was represented by a Gaussian wavepacket of the form

$$\boldsymbol{\psi}(\boldsymbol{R},\boldsymbol{r},\boldsymbol{\theta}) = \left(\frac{\pi^{3/2}}{\sigma_R\sigma_\theta\sigma_r}\right)\mathbf{exp}\left(-\left(\frac{R-R_{eq}}{\sigma_R}\right)^2 - \left(\frac{\theta-\theta_{eq}}{\sigma_\theta}\right)^2 - \left(\frac{r-r_{eq}}{\sigma_r}\right)^2\right) \quad (3)$$

for the equilibrium ground state positions $R_{eq} = 4.99$ au, $\theta_{eq} = 0.0$ rad, and $r_{eq} = 2.2141$ au.[9c] The initial widths were given by the frequencies of each motion $\sigma_R = 0.0022735972$ au, $\sigma_\theta = 0.0010456488$ au, $\sigma_r = 0.0098326$ au.[9a] The wavefunction was assumed to be transferred vertically and instantaneously to the $^1\Pi_1$ excited state at time $\mathrm{t} = 0$ as a result of the UV pump pulse.

The wavepacket was propagated based on the Hamiltonian defined by Eq. (1) in the diabatic representation according to the Split-Operator Fourier Transform (SOFT) method[18] for a total time of 3000 au, discretized in 2 au integration time steps. The variable R was represented in the range $R = [4,12]$ au with 2^8 grid divisions, the angle θ was represented in the range $\theta = [-3/2\,\pi, 3/2\,\pi]$ with 2^8 grid divisions, and the distance r was represented in the range $r = [1,3]$ au with 2^7 grid divisions. The grid size was chosen to ensure that the dynamics was accurately represented. The expectation values of the Jacobi coordinates were recorded every 10 steps. Note that by working in the diabatic representation, problems commonly encountered when working in the adiabatic representation, such as spikes in nonadiabatic couplings vectors or negligence of second-order nonadiabatic couplings, are avoided.

WILEY

X-Ray spectrum

We computed the K-edge X-ray absorption spectrum for a given fixed nuclear configuration by employing the Configuration Interaction Singles approach using the $^1\Pi_1$ electronic wavefunction as the reference state. To obtain this reference state, we employed the Maximum Overlap Method (MOM)[12] as implemented in the Q-Chem package.[19] The MOM strategy consists on applying a Self-Consistent Field (SCF) procedure in which the converged electronic wavefunction maximizes the overlap with the reference state. The reference determinant for the $^1\Pi_1$ valance-excited state was generated by moving one electron from the HOMO to the LUMO, determined at the unrestricted Hartree-Fock (HF) level[20] and DZVP basis set[21] using the GDM algorithm. From the $^1\Pi_1$ state, obtained in this way, we performed CIS calculations with 33 core-valence excitations from the carbon, nitrogen or iodine 1s orbital to obtain the X-ray spectrum at each fixed nuclear configuration.

The above procedure was employed to pre-compute the spectrum for a set of nuclear configurations defined on a regular grid. The grid was 35 × 18 × 7 points in size for the R, θ, and r coordinates respectively, and spanned the range 2.5 Å < R < 6.0 Å, 0 rad < θ < π rad and 1.0 Å < r < 1.3 Å. The spectral lineshape was computed as

$$I(\omega, \boldsymbol{x}) = \sum_k |\mu_k(\boldsymbol{x})|^2 \mathcal{L}(\omega - \omega_k(\boldsymbol{x})) \quad (4)$$

where $\boldsymbol{x}$ represents a fixed ICN nuclear configurations, $|\mu_k|^2$ and ω_k are the transition strength and energy computed by the MOM/CIS scheme, and $\mathcal{L}$ is a Lorentzian broadening with full width at half maximum chosen to approximate the core-hole lifetime broadening of each atom (0.1 eV for C and N; 10.0 eV for I).

The spectrum was recorded in the energy range ω = [280, 310] eV for the C K-edge, ω = [395, 425] eV for the N K-edge, and ω = [31000, 33000] eV for the I K-edge, using 2000 equal divisions. The final transient X-ray spectrum was calculated according to the formula[3f, 14]

$$\sigma(\omega, t) = \int d\boldsymbol{x} |\Psi(\boldsymbol{x}, t)|^2 I(\boldsymbol{x}, t) \quad (5)$$

where $\Psi(\boldsymbol{x}, t)$ is the time-dependent wavefunction associated to the nuclear degrees of freedom. Since the grids representing the wavefunction $\Psi(\boldsymbol{x}, t)$ and the spectral response $I(\omega, \boldsymbol{x})$ are of different size, both regular, linear interpolations were used to evaluate the integral in Eq. (8).

To model the experimental resolution, the spectrum $\sigma(\omega, t)$ was convoluted in energy with Gaussian instrument response functions of different full width at half maximun ($\mathrm{FWHM} = 2\sqrt{2ln2}\Delta\omega$), as follows:

$$\tilde{\sigma}(\omega, t) = \int d\omega' \sigma(\omega', t) e^{-\frac{1}{2}\left[\frac{(\omega-\omega')}{(\Delta\omega)}\right]^2} \quad (6)$$

Natural Transition Orbitals

Natural transition orbitals (NTOs) were obtained by using the Q-Chem package[19] for nuclear configurations sampled from the time-dependent wavepacket. For consistency, NTOs were computed according to the same MOM/CIS scheme applied to compute the X-ray absorption spectra.

Bond order

The I-C bond order of the I-CN bond in the $^1\Pi_1$ excited state was computed from the Natural bond orbitals (NBOs) by using the Q-Chem package.[19] The NBOs are typically localized in two centers and constitute the optimal representation of a Lewis-like molecular bond. That representation enables estimation of the electronic occupancy of each bond, which thus provides a description of the evolution of the I-C sigma bond occupancy. The I-C bond order was computed as a function of time along the photodissociation of ICN in the $^1\Pi_1$ excited state from nuclear configurations sampled from the time-dependent nuclear wavepacket density.

Supplementary Results

Figure S1 shows the Nitrogen and Iodine K-edge TRXAS. The spectra was convoluted with a Gaussian of FWHM 0.5 eV. While the I-C dissociation and the CN vibrations can be captured by the N K-edge TRXAS, the core-hole lifetimes for Iodine are considerably smaller than those of C or N, which produces a broadening in the spectrum that masks the dissociation effect. Note, however, that a weak modulation of the intensity in the I and N TRXAS consistent with the CN vibration can be observed.

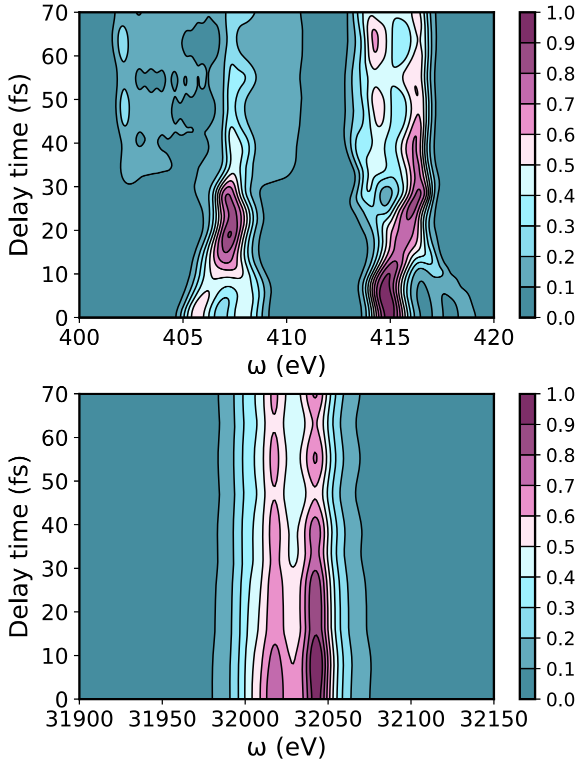

Figure S1: (Top panel) Nitrogen K-edge transient X-ray spectrum of ICN following UV photoexcitation to the $^1\Pi_1$ electronic state. (Bottom panel) Iodine K-edge transient X-ray spectrum of ICN following UV photoexcitation to the $^1\Pi_1$ electronic state.

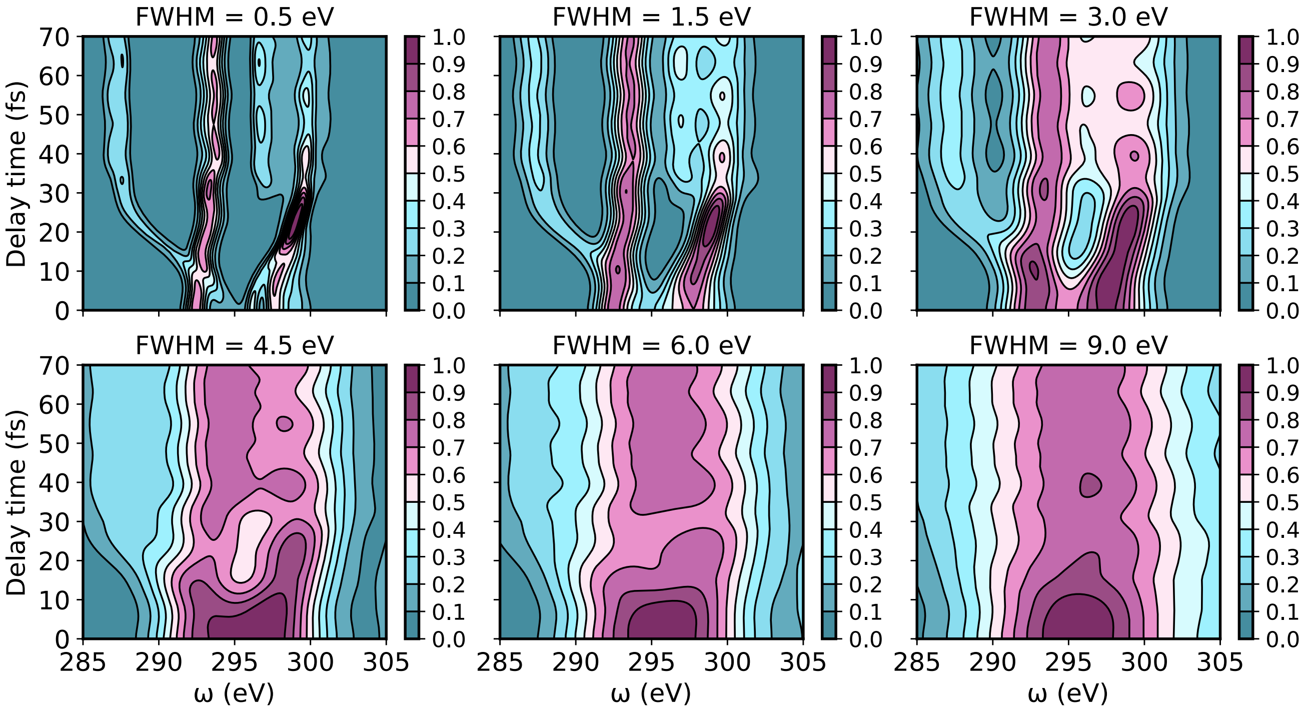

Figure S2: C *K*-edge TRXAS convoluted with Gaussian pulses of different full width half maximum.

Figure S2 (main text) are not convoluted with the instrument resolution function, so they correspond to the infinite resolution TRXAS where the resolution is only limited by the intrinsic lifetimes of the core-hole state. To estimate the experimental resolution limit necessary to capture the transient photochemistry of ICN, we convoluted the spectrum with Gaussian pulses of different width (Figure S2) showing that the I-C photodissociation can be still be captured even at modest resolution as FWHM ~3 eV. Spectral broadening of 4.5 eV gives insufficient resolution to discern the sweeping low-energy peak at 287 eV, although the appearance of a broad shoulder after ~ 20 fs still indicates the completion of the dissociation process.

WILEY

WILEY-VCH
